# Clinical Characteristics and Emergency Response to Sudden Cardiac Arrest/Death Secondary to Cardiomyopathies in Young Athletes

**DOI:** 10.1016/j.jacadv.2026.103093

**Published:** 2026-08-12

**Authors:** Bradley J. Petek, Samantha L. Weller, Timothy W. Churchill, Joseph J. Maleszewski, Randi Delong, Barbara P. Goettsch, Kristen L. Kucera, Kimberly G. Harmon, Jonathan A. Drezner

**Affiliations:** a Sports Cardiology Program, Knight Cardiovascular Institute, Oregon Health & Science University, Portland, Oregon, USA; b Department of Medicine, Oregon Health & Science University, Portland, Oregon, USA; c Division of Cardiology, Massachusetts General Hospital, Boston, Massachusetts, USA; d Cardiovascular Performance Program, Massachusetts General Hospital, Boston, Massachusetts, USA; e Department of Laboratory Medicine & Pathology, Mayo Clinic, Rochester, Minnesota, USA; f Department of Exercise and Sport Science, University of North Carolina at Chapel Hill, Chapel Hill, North Carolina, USA; g Department of Family Medicine and Center for Sports Cardiology, University of Washington, Seattle, Washington, USA

**Keywords:** arrhythmogenic cardiomyopathy, athlete, exercise, hypertrophic cardiomyopathy, sudden cardiac death

## Abstract

**Background:**

Cardiomyopathies (CMs) are a leading cause of sudden cardiac arrest and death (SCA/D) among young competitive athletes.

**Objectives:**

The objective of the study was to describe patient demographics, disease characteristics, circumstances, and resuscitative efforts for athletes with SCA/D related to CMs.

**Methods:**

This observational study identified cases of SCA/D among young competitive athletes aged ≥11 years old competing in middle school, high school, club, college, or semiprofessional/professional sports, and former athletes (within 1 year of participation) through longitudinal surveillance.

**Results:**

Among cases of athlete SCA/D identified from 2002 to 2023 with sufficient information to determine the etiology of SCA/D, 129/454 (28.4%) were attributed to CMs, including 81 (62.8%) hypertrophic CM, 22 (17.1%) arrhythmogenic CM, and 26 (20.2%) with other CMs. Athletes with SCA/D attributed to CMs spanned a wide age range (11-27 years), with hypertrophic CM cases occurring at the youngest average age compared with arrhythmogenic CM or other CM. A total of 87/129 (67.4%) SCA/D events occurred at a sporting facility. Most SCA/D events occurred during practice/training rather than formal games/competition (74.7% vs 25.3%; *P* < 0.0001) and across diverse venues including school and community sporting facilities. Survival from SCA related to CMs was reported in 45/129 (34.9%) of athletes. Survivors of SCA were more likely to receive bystander cardiopulmonary resuscitation (97.6% vs 80.6%; *P* = 0.04) and have on-site automated external defibrillator use (72.4% vs 16.2%; *P* = 0.0001).

**Conclusions:**

SCA/D due to CMs in young athletes occurs across a broad age spectrum and at diverse sports settings. These findings inform funding priorities and resource allocation to optimize emergency preparedness for SCA/D in sport.

Sudden cardiac arrest (SCA) is the leading mechanism of sudden death in young competitive athletes during sports and exercise.^1^, ^2^, ^3^ Inherited cardiomyopathies (CMs) constitute a substantial proportion of SCA and sudden cardiac death (SCD) in athletes, with hypertrophic CM (HCM) consistently identified as one of the leading causes of SCA/D in this population (6% to 36% of cases).^1^, ^2^, ^3^, ^4^, ^5^, ^6^, ^7^, ^8^, ^9^, ^10^, ^11^ Contemporary sports cardiology consensus statements and HCM guidelines now support the possibility of continued competitive sports participation for athletes with HCM and other CMs following a comprehensive evaluation and shared decision-making with an expert clinician.^12^^,^^13^ These recommendations reflect a growing body of evidence demonstrating that exercise and even competitive sports may not uniformly or prohibitively increase the risk of major adverse cardiovascular events in individuals with pathological cardiac conditions.^14^, ^15^, ^16^, ^17^

As the understanding of CM-related SCA/D has advanced, so too has the recognition that outcomes are critically influenced by the immediacy and quality of the cardiac emergency response. Legislative and advocacy initiatives in the United States have accelerated to address this reality. The Cardiomyopathy Health Education, Awareness, Research and Training in Schools (HEARTS) Act (H.R. 6829) was signed into law on December 23, 2024, marking a landmark national effort to enhance cardiac safety in schools. The legislation requires the dissemination of CM education, development of CM risk assessment tools, and formal reporting on CM research. In addition, the HEARTS Act sought to establish a federal grant program to support cardiopulmonary resuscitation (CPR) and automated external defibrillator (AED) training, as well as AED acquisition and maintenance, in public schools. Eligible funding applies to public elementary and secondary schools to cover costs related to emergency action plan (EAP) development, CPR/AED instruction for students, staff, and sports volunteers, and sustained AED accessibility. Although the bill initially passed without dedicated appropriations, advocacy organizations continue to pursue funding to ensure its full implementation.

Parallel state-level efforts, most notably the National Football League (NFL) Smart Heart Sports Coalition launched in 2023, have aimed to improve emergency preparedness standards for high school student-athletes. The coalition’s framework advocates for 3 primary goals: 1) access to an AED at or within 1 to 3 minutes of all athletic venues; 2) an EAP enacted and rehearsed in accordance with national standards and updated annually; and 3) CPR and AED training for coaches.^18^ The NFL Smart Heart Sports Coalition identified states that do not meet these goals to develop state legislation to fill these gaps. More than 25 states have adopted legislation aligning with these benchmarks, yet disparities persist across sports, school districts, and athletic settings. Moreover, several states have passed laws without allocating the necessary funding for implementation. Although these legislative efforts represent significant progress, challenges remain.^19^

The primary aim of this study was to characterize the clinical characteristics and emergency response circumstances among young competitive athletes with SCA/D secondary to CMs. This manuscript focuses on athletes with SCA/D secondary to CMs given the HEARTS act was created with a primary focus on enhancing cardiac safety in schools with a focus on CMs. By linking these findings to current legislative efforts, we aim to inform future legislation and resource allocation for cardiac emergency preparedness in sports.^19^

## Methods

This study was conducted in collaboration with the National Center for Catastrophic Sports Injury Research (NCCSIR) and the University of Washington (UW) Medicine Center for Sports Cardiology. The study was approved by the Institutional Review Board at the University of North Carolina at Chapel Hill and University of Washington. Per ethics approval, our data are not available on request.

### Case identification

Cases of SCA/D were identified using the UW National Collegiate Athletic Association Athlete Death Database (UW-National Collegiate Athletic Association [NCAA]-ADD, SCD cases in college athletes from July 7, 2002-June 30, 2022)^1^ and the NCCSIR Database (SCA/D cases in competitive youth club sports, middle school, high school, college, semiprofessional, professional athletes from July 7, 2014-June 30, 2023).^7^ Complete methods for case identification, study definitions, and adjudication processes have been previously described.^1^^,^^2^^,^^7^ Athletes were identified through multiple independent search strategies including: 1) surveillance of traditional and social media sources; 2) the Parent Heart Watch database; 3) the NCAA Resolutions list; and 4) direct reporting to the NCCSIR, UW Medicine Center for Sports Cardiology, or National Federation of State High School Associations. Duplicate cases were excluded.

### Data collection and etiology of SCA/D adjudication

The data collected for all cases included demographic information, home and school addresses, sports participation, details of the SCA/D event, media reports, and, when available, interviews with the family or school staff, medical history, hospital records, cardiovascular findings, and medical examiner and autopsy reports. All available sources were used to classify race/ethnicity. If medical or postmortem records were not available or did not specify race/ethnicity, media reports and athlete photographs were used to infer this information. Cases in which race/ethnicity could not be determined were classified as unknown.

Adjudicated etiologies of SCA/D were determined through review of all available records using previously published definitions (Supplemental Table 1).^1^^,^^2^^,^^7^ Cases were reviewed by a multidisciplinary panel of physicians with expertise in sports cardiology, sports medicine, and cardiovascular pathology. Discrepancies in interpretation were resolved through panel discussion and unanimous consensus. When multiple potential etiologies were identified, the etiology deemed most likely to have caused the SCA/D event was designated as the primary cause. If several plausible etiologies remained without a clearly identifiable primary cause, the case was classified as “other”, with additional details provided. In instances where the panel’s adjudicated etiology differed from the cause of SCA/D listed on the medical or postmortem records, the adjudicated etiology was used for the present analysis.

Exercise-related SCA/D was defined as events occurring during exercise, whereas nonexercise-related SCA/D was defined as all events occurring during relative rest or sleep, including events <1-hour postexertion.

All available case materials from SCA/D related to CMs were reviewed by at least 1 reviewer (B.J.P./S.L.W.) to determine the circumstances surrounding the SCA/D event (location of SCA/D, training status at the time of SCA/D, exertional status at time of SCA/D, presence/absence of bystander CPR, person performing bystander CPR if applicable, and on-site AED use if available). Bystander CPR was defined as any individual who performed CPR before emergency medical services arrival including potential nonmedical lay responders and on-site health care professionals (eg physicians, nurses, and athletic trainers).

### Inclusion criteria

All competitive athletes aged ≥11 years old participating in middle school, high school, club, college, or semiprofessional/professional sports, as well as former athletes (within 1 year of cessation from competitive sports) who suffered SCA/D and were identified by the UW-NCAA-ADD and NCCSIR were considered for study inclusion. Athletes were included in the present study if a CM was determined to be the underlying etiology of the SCA/D event.

CM cases were defined as SCA/D related to HCM, arrhythmogenic CM (ACM), or other CMs (CM, including left ventricular noncompaction CM, restrictive CM, dilated CM, and CM not otherwise specified). Idiopathic left ventricular hypertrophy was excluded from this analysis, as prior studies suggest that this postmortem finding is phenotypically distinct from HCM and may be associated with a range of cardiac pathologies, including arrhythmic syndromes, when genetic testing of first-degree relatives is performed.^20^

### Statistical analysis

Standard descriptive statistics were used to characterize demographic information. Categorical variables are presented as frequencies (n) and percentages (%), and continuous variables are reported as means and SDs. Differences in the mean age of SCA/D across CM types were assessed using an analysis of variance test. Two-proportion chi-square tests or Fisher exact tests, as appropriate, were used to assess differences between sex and race/ethnicity among CM types; to compare athletes with SCA who survived vs SCD with respect to factors related to emergency preparedness (witnessed arrest, bystander CPR, and on-site AED use); to assess differences related to the type of training or competition at the time of SCA/D; and to compare athletes with SCA who survived vs SCD by location of death. A *P* < 0.05 was considered statistically significant. Statistical analyses were performed using R: A Language and Environment for Statistical Computing (R Core Team).

## Results

A total of 742 SCA/D cases were identified during the 21-year study period, consisting of 427 SCD cases and 315 SCA cases with survival. Among the 427 athletes who suffered SCD, sufficient information was available to determine an etiology in 326/427 cases (76.4%). Among the 315 athletes who experienced SCA and survived, an etiology could be established in 128/315 cases (40.6%). Overall, 454/742 (61.2%) athletes had an identified etiology associated with their SCA/D. Of these, 129/454 (28.4%) were determined to have an underlying CM (Figure 1), including 45/129 athletes (34.9%) with SCA who survived and 84/129 athletes (65.1%) with SCD (Central Illustration).

**Figure 1:**
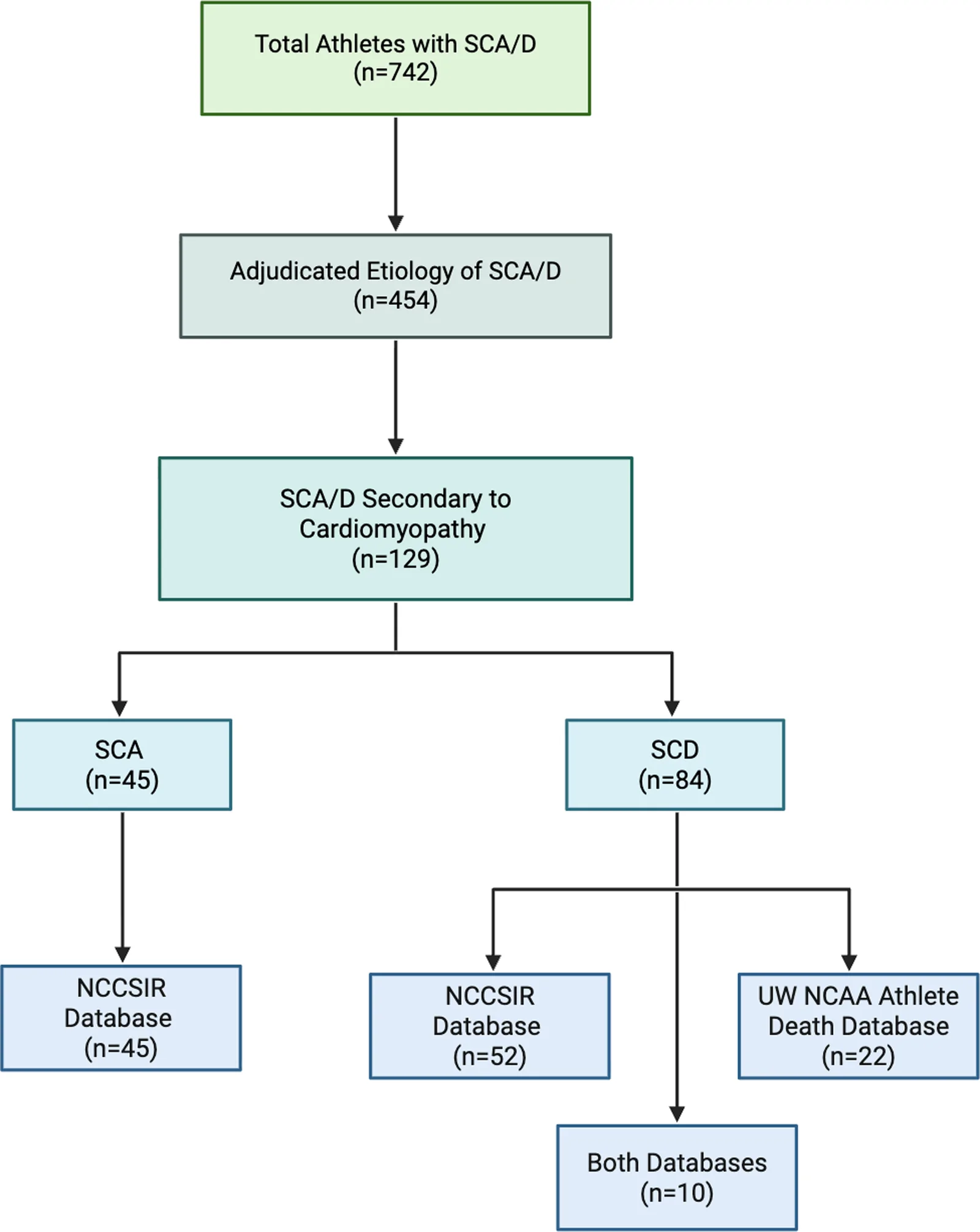
Flow Diagram for Case Identification and Inclusion NCAA = National Collegiate Athletic Association; NCCSIR = National Center for Catastrophic Sports Injury Research; SCA = sudden cardiac arrest; SCA/D = sudden cardiac arrest/death; SCD = sudden cardiac death; UW = University of Washington A flow diagram for case inclusion from the NCCSIR and UW NCAA databases is presented.

**Central Illustration:**
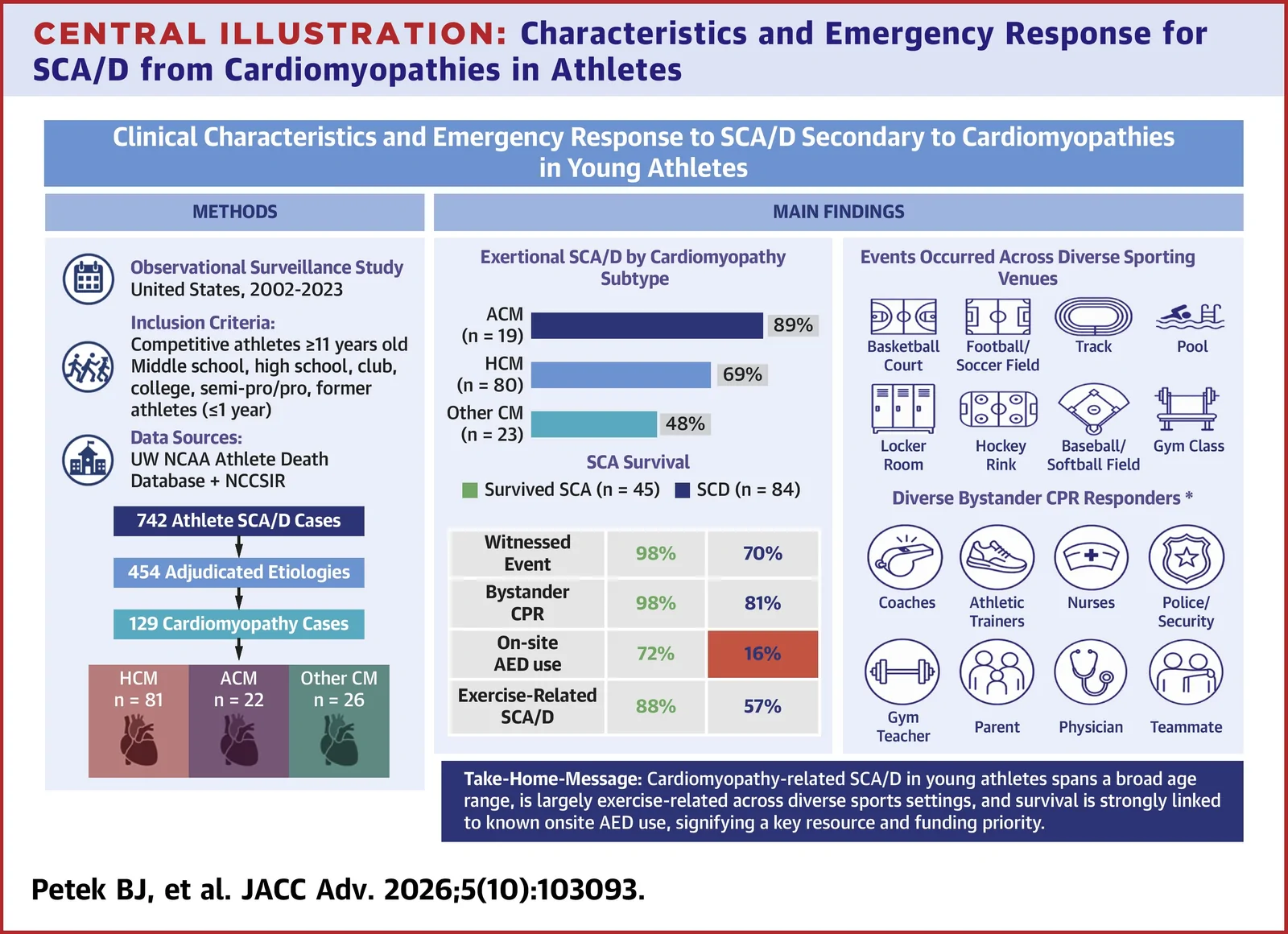
Characteristics and Emergency Response for SCA/D from Cardiomyopathies in Athletes The central illustration depicts the study title, methods, major results, and conclusions. AED = automated external defibrillator; CM = cardiomyopathy; CPR = cardiopulmonary resuscitation; ACM = arrhythmogenic cardiomyopathy; HCM = hypertrophic cardiomyopathy; NCAA = National Collegiate Athletic Association; NCCSIR = National Center for Catastrophic Sport Injury Research; SCA = sudden cardiac arrest; SCA/D = sudden cardiac arrest/death; SCD = sudden cardiac death; UW = University of Washington. ∗Bystander CPR responder was defined as any individual who performed CPR prior to arrival of emergency medical services

### Athlete characteristics

An overview of athlete characteristics is presented in Table 1. There were statistically significant differences in the age of individuals with SCA/D secondary to HCM (age 16.9 ± 2.6 years, range 12-27 years), ACM (age 19.1 ± 3.2 years, range 15-26 years), and other CM (age 18.3 ± 3.5 years, range 11-24 years, *P* = 0.004). Overall, the cohort was predominantly males (89.1%), with no significant differences in sex observed between CM types. Athletes with SCA/D secondary to HCM were more likely identified as Black compared to athletes with ACM (51.9% vs 18.2%; *P* = 0.007) and other CM (51.9% vs 19.2%; *P* = 0.007). The most common level of sport participation was high school (48.1%) followed by collegiate (33.3%) and middle school athletics (10.1%). Basketball was the most frequently involved sport (36.4%), followed by football (26.4%) and baseball (7.8%).

**Table 1:** Demographics, Methods of Case Adjudication, and Sudden Cardiac Arrest/Death Characteristics Stratified by Type of Cardiomyopathy

	All CM (N = 129)	HCM (n = 81)	ACM (n = 22)	Other CM (n = 26)^a^
Baseline demographics				
Age, y	17.6 (3.0)	16.9 (2.6)	19.1 (3.2)	18.3 (3.5)
Female	14 (10.9)	7 (8.6)	4 (18.2)	3 (11.5)
Male	115 (89.1)	74 (91.4)	18 (81.8)	23 (88.5)
White	71 (55.0)	33 (40.7)	18 (81.8)	20 (76.9)
Black	51 (39.5)	42 (51.9)	4 (18.2)	5 (19.2)
Other race	7 (5.4)	6 (7.4)	0 (0.0)	1 (3.8)
Current level of competition				
Middle school	13 (10.1)	10 (12.3)	0 (0.0)	3 (11.5)
High school	62 (48.1)	45 (55.6)	10 (45.5)	7 (26.9)
College	43 (33.3)	22 (27.2)	7 (31.8)	14 (53.8)
Semipro/professional	3 (2.3)	1 (1.2)	2 (9.1)	0 (0.0)
Club sports	3 (2.3)	1 (1.2)	1 (4.5)	1 (3.8)
Former athlete	5 (3.9)	2 (2.5)	2 (9.1)	1 (3.8)
Primary sport				
Baseball	10 (7.8)	4 (4.9)	1 (4.5)	5 (19.2)
Basketball	47 (36.4)	34 (42.0)	5 (22.7)	8 (30.8)
Football	34 (26.4)	24 (29.6)	3 (13.6)	7 (26.9)
Soccer	8 (6.2)	5 (6.2)	2 (9.1)	1 (3.8)
Track and field	5 (3.9)	2 (2.5)	0 (0.0)	3 (11.5)
Other	25 (19.4)	12 (14.8)	11 (50.0)	2 (7.7)
Records available for case adjudication				
Autopsy report	57 (44.2)	33 (40.7)	9 (40.9)	15 (57.7)
Media report	48 (37.2)	35 (43.2)	9 (40.9)	4 (15.4)
Coroner’s report/COD	13 (10.1)	7 (8.6)	1 (4.5)	5 (19.2)
Interview	7 (5.4)	3 (3.7)	2 (9.0)	2 (7.7)
Medical records	4 (3.1)	3 (3.7)	1 (4.5)	0 (0.0)
Type of SCA/D event				
SCA	45 (34.9)	30 (37.0)	10 (45.5)	5 (19.2)
SCD	84 (65.1)	51 (63.0)	12 (54.5)	21 (80.8)

Values are average (SD) or n (%) accordingly.

ACM = arrhythmogenic cardiomyopathy; CM = cardiomyopathy; COD = cause of death; HCM = hypertrophic cardiomyopathy; SCA = sudden cardiac arrest; SCA/D = sudden cardiac arrest/death; SCD = sudden cardiac death.

a Included 13 athletes with cardiomyopathy not otherwise specified, 9 athletes with dilated cardiomyopathy, 2 athletes with left ventricular noncompaction cardiomyopathy, and 2 athletes with restrictive cardiomyopathy.

### Circumstances and emergency medical response following SCA/D

An overview of the circumstances surrounding SCA/D events and the corresponding emergency medical responses are presented in Table 2. Although all elements of the event and emergency response were not known in some cases, comparisons were made given the available information. Compared with athletes who suffered SCD, survivors of SCA were more likely to have an exercise-related SCA/D event (88.4% vs 57.0%; *P* = 0.0008), witnessed arrest (97.8% vs 69.7%; *P* = 0.0005), receive bystander CPR (97.6% vs 80.6%; *P* = 0.04), and known on-site AED use (72.4% vs 16.2%; *P* = 0.0001).

**Table 2:** Circumstances and Emergency Medical Response for Sudden Cardiac Arrest/Death

	Total SCA (N = 45)	Total SCD (n = 84)	Sporting Facility SCA (n = 38)	Sporting Facility SCD (n = 49)
Exertional status				
Exercise-related SCA/D	38 (84.4)	45 (53.6)	36 (94.7)	42 (85.7)
SCA/D <1 h postexertion	3 (6.7)	7 (8.3)	2 (5.3)	5 (10.2)
Relative rest, but awake	2 (4.4)	13 (15.5)	0 (0.0)	2 (4.1)
Sleep	0 (0.0)	14 (16.7)	0 (0.0)	0 (0.0)
Unknown	2 (4.4)	5 (6.0)	0 (0.0)	0 (0.0)
Bystander presence				
Witnessed arrest	44 (97.8)	53 (63.1)	38 (100)	47 (95.9)
Unwitnessed arrest	1 (2.2)	23 (27.4)	0 (0.0)	2 (4.1)
Unknown	0 (0.0)	8 (9.5)	0 (0.0)	0 (0.0)
Bystander CPR				
Bystander CPR performed	40 (97.8)	29 (34.5)	34 (89.5)	22 (44.9)
No bystander CPR	1 (2.2)	7 (8.3)	1 (2.6)	4 (8.2)
Unknown	4 (8.9)	48 (57.1)	3 (7.9)	23 (46.9)
AED use				
AED use on-site	21 (46.7)	6 (7.1)	19 (50.0)	5 (10.2)
No AED use	8 (17.8)	31 (36.9)	6 (15.8)	10 (20.4)
Unknown	16 (35.6)	47 (56.0)	13 (34.2)	34 (69.4)
Location of arrest				
School	38 (84.4)	37 (44.0)	34 (89.5)	34 (69.4)
Public recreation area	4 (8.8)	13 (15.5)	4 (10.5)	13 (26.5)
Home	1 (2.2)	22 (26.2)	0 (0.0)	0 (0.0)
Other^a^	1 (2.2)	4 (4.8)	0 (0.0)	2 (4.1)
Unknown	1 (2.2)	8 (9.5)	0 (0.0)	0 (0.0)
Type of exercise				
Game/competition	15 (39.5)	7 (14.0)	15 (39.5)	7 (14.3)
Practice/training	19 (50.0)	34 (68.0)	19 (50.0)	33 (67.3)
Pick-up game	0 (0.0)	6 (12.0)	0 (0.0)	6 (12.2)
Gym class	4 (10.5)	3 (6.0)	4 (10.5)	3 (6.1)
Nonexertional	4 (9.1)	25 (29.8)	0 (0.0)	0 (0.0)
Hiking trail	0 (0.0)	1 (1.2)	0 (0.0)	0 (0.0)
Unknown	8 (9.5)	2 (4.5)	0 (0.0)	0 (0.0)

AED = automated external defibrillator; CPR = cardiopulmonary resuscitation; other abbreviations as in Table 1.

There were 3 athletes in the total SCD group where there was an AED available, but use was unknown, and 3 athletes in the sporting facility SCD group where there was an AED available, but use was unknown.

a The other group included total SCA = 1 car, total SCD = 1 hiking trail, 1 car, 2 basketball court (unknown if school or community court), sporting facility SCD = 2 basketball court (unknown if school or community court).

A total of 87/129 (67.4%) SCA/D events occurred at a sporting facility (68 school sporting facilities, 17 public recreation areas, and 2 basketball courts where the setting, either school or public, could not be determined). Most SCA/D events at sporting facilities occurred during practice/training (including team practices, pick-up basketball, and gym classes) compared to a formal game/competition (74.7% vs 25.3%; *P* < 0.0001).

Survival rates for SCA in athletes with CMs varied by setting: 34/68 (50.0%) athletes survived SCA events occurring at school sporting facilities, compared with 4/17 (23.5%) athletes at public recreation facilities (*P* = 0.09). Four SCA events occurred during sanctioned club sports at school sporting facilities (2 in school basketball gyms, 1 at a school pool, and 1 on a school lacrosse field), with 2/4 (50%) athletes surviving.

Across SCA/D events known to occur at school or public recreation facilities (n = 85), the specific on-site locations varied widely (Figure 2). In addition, among SCA/D events occurring at sporting facilities in which the individuals performing bystander CPR were known (n = 40), coaches were most frequently involved in CPR (40.0%), followed by athletic trainers (35.0%), and nurses (15.0%) (Figure 3). When limited to SCA/D events occurring in athletes competing at the middle school and high school levels with known bystander CPR providers (n = 28), coaches remained the most common responders (46.4%), followed by athletic trainers (25.0%) and school nurses (17.9%).

**Figure 2:**
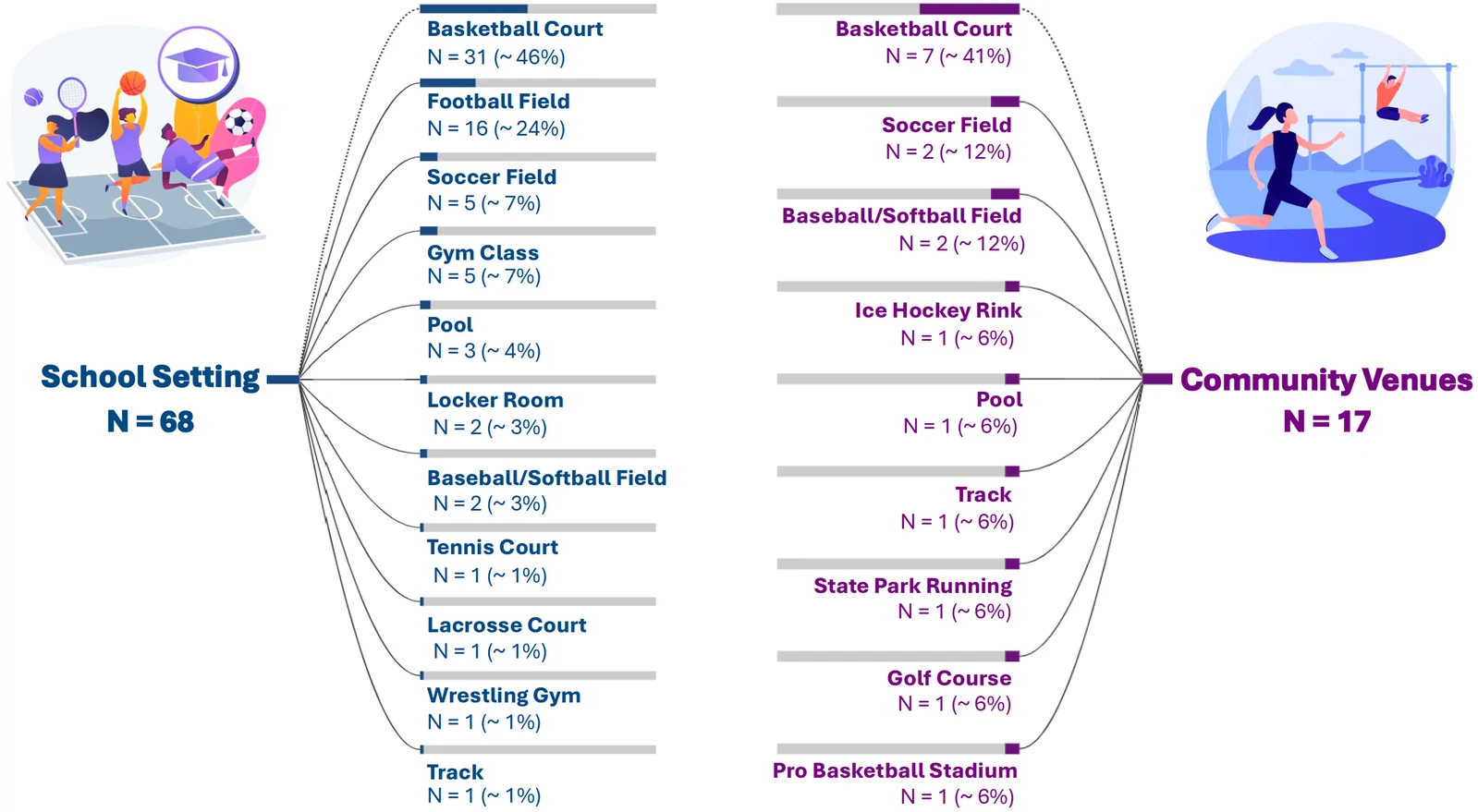
Locations of Sudden Cardiac Arrest/Death at School vs Community Sporting Facilities Blue bars = percentage of events occurring at a known location at school sporting facilities. Purple bars = percentage of events occurring at a known location at community venues with sporting facilities.

**Figure 3:**
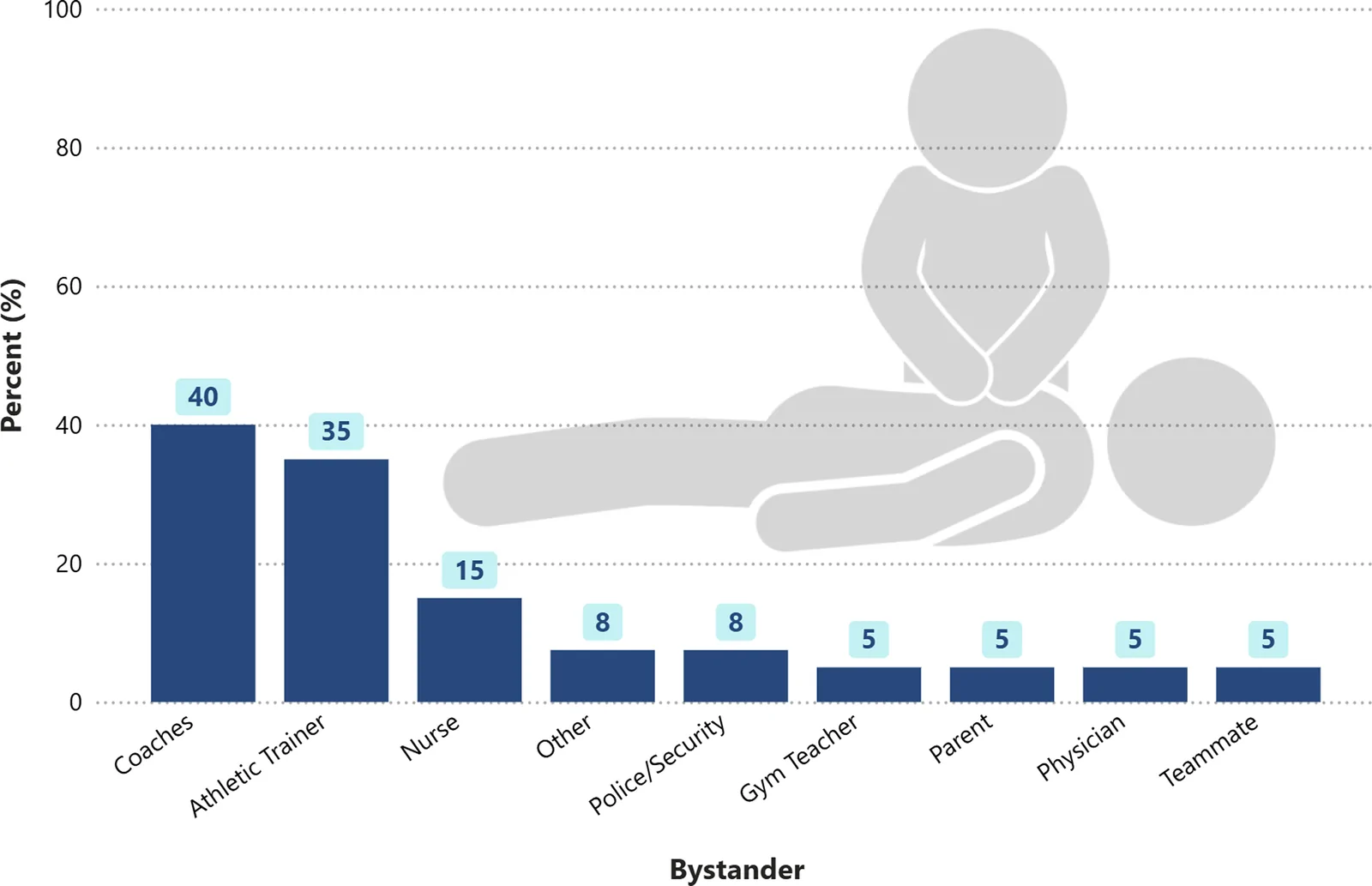
Composition of Individuals Performing Bystander Cardiopulmonary Resuscitation at Sporting Facilities (n = 40) Blue bars = percentage of SCA/D events with that type of bystander performing CPR.

With respect to athletes with known exertional status at the time of SCA/D, 17/19 (89.5%) athletes with ACM experienced SCA/D during exercise, compared to 55/80 (68.8%) athletes with HCM and 11/23 (47.8%) with other CM (Figure 4).

**Figure 4:**
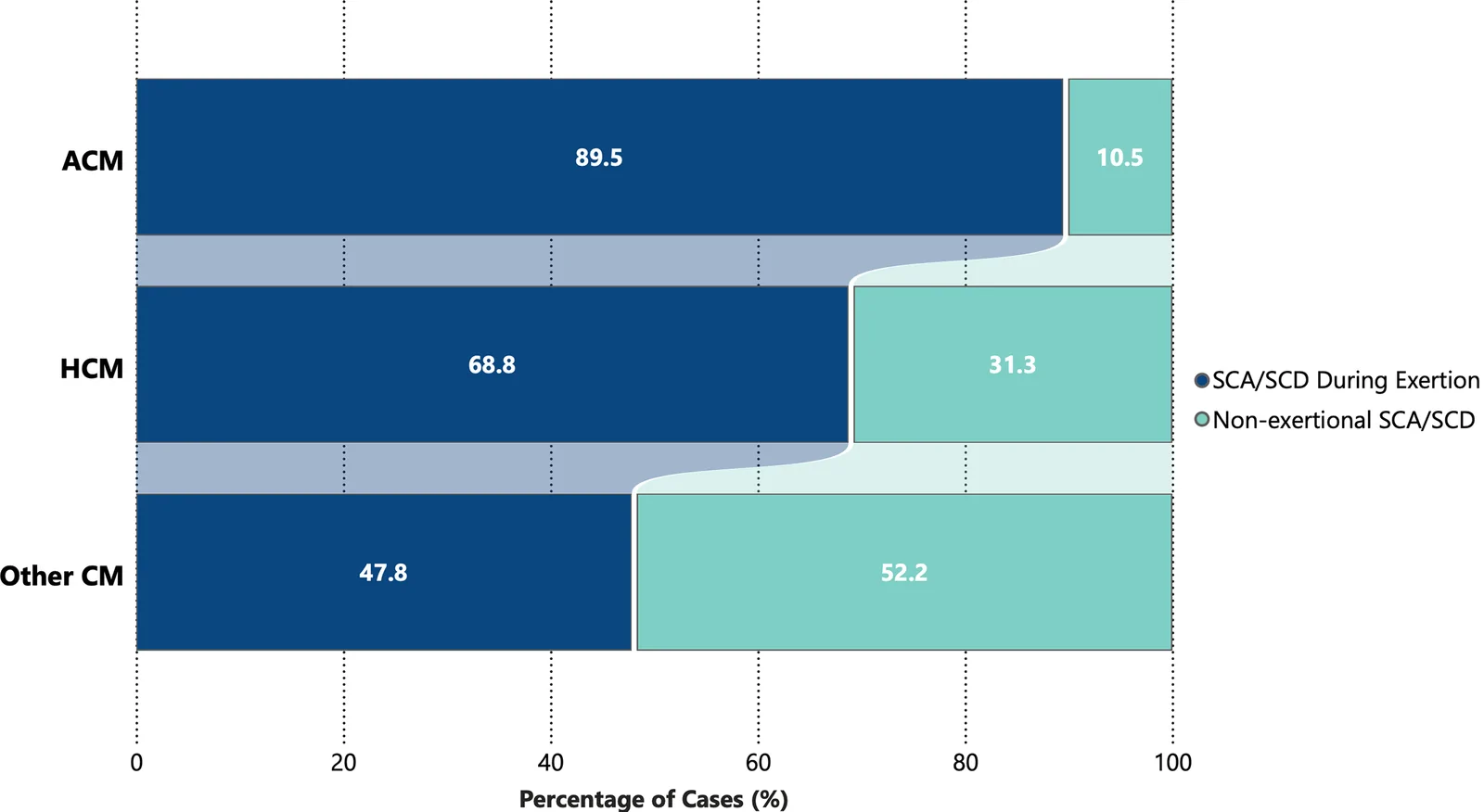
Exertional Status at Time of SCA/D by Cardiomyopathy Type ACM = arrhythmogenic cardiomyopathy; Blue bars = percentage of SCA/D cases occurring during exertion; CM = cardiomyopathy; Green bars = percentage of nonexertional SCA/D cases; HCM = hypertrophic cardiomyopathy; other abbreviations as in Figure 1.

## Discussion

SCA/D in young athletes with underlying CMs remains a complex problem at the intersection of inherited disease, exertional physiology, and variable emergency preparedness. In this national, longitudinal surveillance study, we identified several key findings. First, athletes with CM-related SCA/D occurred across a wide age spectrum (11-27 years), underscoring the need for robust emergency preparedness even in younger athletic settings, including middle schools. Second, athletes who survived CM-related SCA were more likely to have had a witnessed SCA event, receive bystander CPR, and have an on-site AED used, consistent with well-established factors that impact survival from SCA in the general population.^21^ Third, most SCA/D events occurred during practice/training rather than a formal game/competition and took place across a wide variety of venues spanning both school-based and public facilities. Fourth, bystander CPR was delivered by individuals with wide-ranging backgrounds (coaches, athletic trainers, teammates, security guards, gym teachers, family members, nurses, physicians, and school staff). Finally, exercise-related SCA/D was not uniform across CM types, with athletes with ACM experiencing the highest proportion of exercise-related events (89.5%), followed by HCM (68.8%) and other CM (47.8%).

Both the HEARTS Act and NFL Smart Heart Coalition have advocated for legislation and ongoing efforts to strengthen cardiac emergency preparedness in schools, with the focus on AED access, CPR training and structured EAPs in school settings. The present study provides empirical support for these initiatives by demonstrating that CM-related SCA/D occurs across the full youth athletic spectrum, including middle school athletes. In particular, the NFL Smart Heart Coalition’s recommendation for AED access within 1 to 3 min of all athletic activities is reinforced by our findings, as SCA/D among athletes with CM occurred across an array of locations on school campuses, including practice fields, gyms, and other ancillary training spaces. Our findings highlight both the importance and challenge of implementing this goal. In addition, the NFL Smart Heart Coalition recommends CPR and AED training for all coaches. Although our data confirm that coaches frequently serve as first responders, they also show that a wide range of individuals, including teachers, athletic trainers, teammates, family members, and other community members, often initiate resuscitation. This highlights an important gap in current legislative policy. Limiting CPR and AED training to coaches does not adequately prepare the broader group of responders who are commonly involved in real-world events. Expanding CPR and AED education to all participants and regular spectators of youth sports would better reflect the true landscape of emergency response.^22^

Current legislation is focused almost entirely on school-based environments, yet a substantial number of SCA events in this study occurred at public recreation venues such as parks and community fields, where AED access and emergency planning are inconsistent. Survival appeared to be lower in these settings, suggesting a potential area of vulnerability for young athletes. These findings emphasize the need for future advocacy and legislation that extends cardiac emergency preparedness standards to public sporting venues and to athletic activities that occur outside of school oversight, to ensure equitable protection for all young athletes regardless of where they train or compete.^22^

The American College of Cardiology/American Heart Association consensus statement for competitive athletes with cardiovascular abnormalities provides varying recommendations for sports participation based on the underlying etiology of CMs in young athletes.^12^ The document states that it is “reasonable to consider” competitive sports participation for athletes with HCM and dilated CM after a comprehensive expert assessment with shared-decision making that incorporates individualized risks and benefits, including the risk for SCD. In contrast, the statement concludes that the “risks likely outweigh the benefits” for competitive sports participation among athletes with plakophilin-2 gene ACM, reflecting prior evidence that exercise may accelerate disease progression and increase arrhythmic vulnerability in this genotype. Most existing evidence regarding exercise-related risk in plakophilin-2 gene ACM has been derived from retrospective patient-reported endurance activity. These approaches, although informative, may not fully capture the real-world athletic exposures that influence arrhythmic events.^23^, ^24^, ^25^, ^26^, ^27^ The current study demonstrated that athletes with ACM exhibited the highest proportion of exercise-related SCA/D (89.5%). Although phenotypes and genotypes were not available for detailed analysis, the observed pattern may support a potential heightened exertional vulnerability in this population. Although most athletes spend the minority of their waking hours exercising (usually limited to a few hours per day), this analysis has significant limitations without comparison to a control group. Prospective monitoring of physical activity in all ACM genotypes is needed to improve risk assessment for athletes.^10^^,^^23^

### Study limitations

This study has limitations that warrant further discussion. First, because there is no mandatory reporting system for SCA/D events in young athletes in the United States, some cases may not have been captured. Second, there may be inherent bias in case capture given that cases occurring in public locations may be more likely to be reported in media reports than SCA/D events that occur at home. Third, details related to the emergency response, including the provision of bystander CPR and the use of an AED, were missing in a substantial portion of cases (Table 2). This limits the precision with which associations between emergency preparedness and survival can be interpreted. With growing national efforts to improve outcomes following SCA, the establishment of a mandatory reporting structure with resuscitation characteristics should be considered in order to enhance epidemiologic accuracy. Fourth, the precise cardiovascular etiology of SCA/D could not be determined in nearly one-quarter of the SCD cases and more than half of the SCA cases, which may have influenced the distribution of CM types and the analysis of exertional patterns. In addition, the diagnosis of CMs can be difficult in both the living and postmortem settings, and there is also no standardized evaluation process for cardiovascular autopsies among young athletes in the United States who have a SCD event, which may affect results and remains an important future quality initiative.^28^ For transparency, we have included the method for CM case adjudication in Table 1. Fifth, because data were obtained from multiple sources with heterogeneous case identification and capture methods, detailed clinical and phenotypic information was not uniformly available, limiting the ability to draw definitive conclusions about genotype or disease severity. Sixth, the combined use of two large surveillance systems, one capturing both SCA and SCD events (NCCSIR) and the other capturing only SCD events (UW-NCAA-ADD), may have affected comparative analyses. Seventh, incidence of SCA/D could not be accurately determined in the current study given there is no accurate denominator of sports participation in the large range of athlete categories from the NCCSIR data. Therefore, trends of SCA/D over time could not be assessed. A better understanding of trends of SCA/D incidence in young competitive athletes, and any reasons for changes in incidence (eg emergency preparedness, preparticipation screening, and/or eligibility/disqualification decisions), remain important areas for future research. Finally, race was determined through available records and in some instances through media descriptions or photographs. Race is a social rather than biologic construct,^29^ and individuals may identify differently from the classification used in this study. Furthermore, athletes who identify as more than 1 race may not have been accurately represented.

## Conclusions

A prompt cardiac emergency response is essential to improving outcomes for athletes who experience SCA due to CMs. These events occur across a wide age range and array of sporting environments, including school facilities and public recreation spaces, and resuscitation efforts are initiated by individuals with diverse roles in the athletic community. In addition, exertional risk may vary by CM type, suggesting distinct vulnerability profiles that warrant further investigation. These findings support current legislative efforts aimed at improving outcomes among individuals and athletes with SCA/D but also reinforce the need for expanded efforts that encompass all public sporting venues. Broader access to AEDs and widespread CPR training for all individuals who participate in or regularly attend youth sports are essential steps toward improving outcomes and ensuring equitable protection for all young athletes.

Perspectives **COMPETENCY IN PATIENT CARE AND SYSTEMS-BASED PRACTICE:** The current study demonstrated that SCA/D due to CMs in young athletes occurs across a broad age spectrum, are largely exercise-related at diverse sporting venues, and survival is associated with bystander CPR and on-site AED use provided by a range of individuals.

**TRANSLATIONAL OUTLOOK:** These findings support current legislative efforts aimed at improving outcomes among individuals and athletes with SCA/D but also reinforce the need for expanded efforts to improve emergency preparedness at all public sporting venues and for broader access to AEDs and widespread CPR training for all individuals who participate in or regularly attend youth sports.

## Funding sources and author disclosures

Dr Petek’s Sports Cardiology Program has received compensation for preparticipation cardiovascular screening for the National Hockey League Scouting Combine, University of Portland, and Portland State University. In addition, his Sports Cardiology Program has received funding from the Joel Cornette Foundation for the Outcomes Registry for Cardiac Conditions in Athletes study. Dr Churchill has received compensation for his role as team cardiologist for the Boston Bruins. Dr Drezner has received funding from the 10.13039/100020506American Medical Society for Sports Medicine, the 10.13039/100000968American Heart Association, and the NCCSIR. Dr Harmon has received funding from the Football Research, Inc, and the 10.13039/100000968American Heart Association; and she is employed as the Deputy Chief Medical Officer of the National Hockey League (NHL). The views and opinions expressed are solely those of the authors and do not necessarily represent those of the NHL. Dr Kucera is supported by funds from the NCCSIR which is funded by 10.13039/100012676National Collegiate Athletic Association (NCAA), the National Federation of State High School Associations (NFHS), the National Athletic Trainers’ Association (NATA), the National Operating Committee on Standards for Athletic Equipment (NOCSAE), and the American Medical Society for Sports Medicine (AMSSM). All other authors have reported that they have no relationships relevant to the contents of this paper to disclose.

## References

[1] Petek B.J., Churchill T.W., Moulson N. (2024). Sudden Cardiac death in National Collegiate Athletic Association athletes: a 20-year study. Circulation.

[2] Peterson D.F., Kucera K., Thomas L.C. (2020). Aetiology and incidence of sudden cardiac arrest and death in young competitive athletes in the USA: a 4-year prospective study. Br J Sports Med.

[3] Harmon K.G., Asif I.M., Maleszewski J.J. (2015). Incidence, cause, and comparative frequency of sudden cardiac death in National Collegiate Athletic Association athletes: a decade in review. Circulation.

[4] Corrado D., Basso C., Rizzoli G., Schiavon M., Thiene G. (2003). Does sports activity enhance the risk of sudden death in adolescents and young adults?. J Am Coll Cardiol.

[5] Finocchiaro G., Papadakis M., Robertus J.-L. (2016). Etiology of sudden death in sports. J Am Coll Cardiol.

[6] Maron B.J., Haas T.S., Ahluwalia A., Murphy C.J., Garberich R.F. (2016). Demographics and epidemiology of sudden deaths in young competitive athletes: from the United States national registry. Am J Med.

[7] Petek B.J., Churchill T.W., Moulson N. (2025). Survival outcomes after sudden cardiac arrest in young competitive athletes from the United States. J Am Coll Cardiol.

[8] Holst A.G., Winkel B.G., Theilade J. (2010). Incidence and etiology of sports-related sudden cardiac death in Denmark—Implications for preparticipation screening. Heart rhythm.

[9] Maron B.J., Haas T.S., Ahluwalia A., Rutten-Ramos S.C. (2013). Incidence of cardiovascular sudden deaths in Minnesota high school athletes. Heart Rhythm.

[10] Maron B.J., Haas T.S., Duncanson E.R., Garberich R.F., Baker A.M., Mackey-Bojack S. (2016). Comparison of the frequency of sudden cardiovascular deaths in young competitive athletes versus nonathletes: should we really screen only athletes?. Am J Cardiol.

[11] Harmon K.G., Asif I.M., Maleszewski J.J. (2016). Incidence and etiology of sudden cardiac arrest and death in high school athletes in the United States. Mayo Clin Proc.

[12] Kim J.H., Baggish A.L., Levine B.D. (2025). Clinical considerations for competitive sports participation for athletes with cardiovascular abnormalities: a scientific statement from the American Heart Association and American College of Cardiology. J Am Coll Cardiol.

[13] Ommen S.R., Ho C.Y., Asif I.M. (2024). 2024 AHA/ACC/AMSSM/HRS/PACES/SCMR Guideline for the management of hypertrophic cardiomyopathy: a report of the American Heart Association/American College of Cardiology Joint Committee on clinical practice guidelines. J Am Coll Cardiol.

[14] Lampert R., Ackerman M.J., Marino B.S. (2023). Vigorous exercise in patients with hypertrophic cardiomyopathy. JAMA Cardiol.

[15] Basu J., Nikoletou D., Miles C. (2025). High intensity exercise programme in patients with hypertrophic cardiomyopathy: a randomized trial. Eur Heart J.

[16] Saberi S., Wheeler M., Bragg-Gresham J. (2017). Effect of moderate-intensity exercise training on peak oxygen consumption in patients with hypertrophic cardiomyopathy: a randomized clinical trial. JAMA.

[17] Martinez K.A., Bos J.M., Baggish A.L. (2023). Return-to-Play for elite athletes with genetic heart diseases predisposing to sudden cardiac death. J Am Coll Cardiol.

[18] Dineen E.H., Lawrence M., Husaini M. (2024). Advocacy in sports cardiology. JACC: Adv.

[19] Petek B.J., Drezner J.A. (Feb 25 2026). Emergency preparedness for sudden cardiac arrest in young athletes: time to adjust the goalposts. J Am Coll Cardiol.

[20] Finocchiaro G., Dhutia H., Gray B. (2020). Diagnostic yield of hypertrophic cardiomyopathy in first-degree relatives of decedents with idiopathic left ventricular hypertrophy. EP Europace.

[21] Del Rios M., Bartos J.A., Panchal A.R. (2025). Part 1: executive summary: 2025 American Heart Association Guidelines for cardiopulmonary resuscitation and emergency cardiovascular care. Circulation.

[22] Petek B.J., Drezner J.A. (2026). Emergency preparedness for sudden cardiac arrest in young athletes. JACC.

[23] James C.A., Bhonsale A., Tichnell C. (2013). Exercise increases age-related penetrance and arrhythmic risk in arrhythmogenic right ventricular Dysplasia/Cardiomyopathy–Associated desmosomal mutation carriers. J Am Coll Cardiol.

[24] Ruwald A.-C., Marcus F., Estes N.A.M. (2015). Association of competitive and recreational sport participation with cardiac events in patients with Arrhythmogenic Right Ventricular Cardiomyopathy: results from the North American Multidisciplinary study of Arrhythmogenic Right Ventricular Cardiomyopathy. Eur Heart J.

[25] Saberniak J., Hasselberg N.E., Borgquist R. (2014). Vigorous physical activity impairs myocardial function in patients with arrhythmogenic right ventricular cardiomyopathy and in mutation positive family members. Eur J Heart Fail.

[26] Sawant A.C., te Riele A.S.J.M., Tichnell C. (2016). Safety of American Heart Association-recommended minimum exercise for desmosomal mutation carriers. Heart Rhythm.

[27] Lie Ø.H., Dejgaard L.A., Saberniak J. (2018). Harmful effects of exercise intensity and exercise duration in patients with arrhythmogenic cardiomyopathy. JACC Clin Electrophysiol.

[28] Petek B.J., Maleszewski J.J., Harmon K.G. (2025). Post-mortem evaluation of young competitive athletes following sudden cardiac death: time to shock the system?. Circulation.

[29] Krishnan S., Guseh J.S., Chukumerije M. (2024). Racial disparities in sports cardiology: a review. JAMA Cardiol.

