# Supplementary material for: Clinical Characteristics and Emergency Response to Sudden Cardiac Arrest/Death Secondary to Cardiomyopathies in Young Athletes
Source: JACC Adv. 2026 Aug 12;5(10):103093. doi: 10.1016/j.jacadv.2026.103093 (PMC13699712; doi:10.1016/j.jacadv.2026.103093)
Supplement: Supplementary file 1 [file mmc1.pdf]

SUPPLEMENTAL MATERIAL

Table of Contents

Supplemental Tables.. 2-3
Supplemental Table 1. Guidelines for Etiologies of Sudden Cardiac Death................................ 2-3

Supplemental Table 1. Guidelines for Etiologies of Sudden Cardiac Death

Hypertrophic Cardiomyopathy

• Heart weight ≥50% of expected mean based on sex, age, and body size for weight (using the Mayo nomograms)[1,2] plus at least one of the following:
- Histological myocyte disarray
- Septal mitral valve contact lesion (implying systolic anterior motion of the anterior mitral valve leaflet)
- Asymmetrical LV hypertrophy, particularly ventricular septal – left ventricular free wall ratio ≥1.3

• Significant (>75% of the area of a section) myocyte disarray in a basal or midventricular section but not meeting weight criteria.

Arrhythmogenic Cardiomyopathy

• Gross fibrofatty replacement of either ventricular free wall (excluding anterior RV in older individuals)
• The fatty change should appear “infiltrative” with a perpendicular pattern to the epicardial surface
• Variable degrees of fibrosis, vacuolization, and lymphocytic myocarditis

Idiopathic LVH/possible cardiomyopathy

• Heart weight ≥50% of the expected mean based on sex, age, and body size for weight (using the Mayo nomograms)[1,2]
• Heart weight ≤50% of the expected mean based on sex, age, and body size for weight (using Mayo nomograms)[1,2], but with:
- Features suggestive (but not diagnostic) of CM, including: LV wall >16 mm, interstitial fibrosis (non-replacement type), significant histological myocyte hypertrophy
- No specific features of CM

Cardiomyopathy NOS

• Heart weight does not meet weight criteria
• There are histological changes such as hypertrophy or fibrosis
• No/ minimal myocyte disarray not meeting criteria for HCM
• Does not meet criteria for DCM
• No pathological features suggestive of HCM

Dilated cardiomyopathy

• Heart weight ≥50% expected mean based on sex, age, and body size for weight (using the Mayo nomograms)[1,2] without myocyte disarray
- LV wall <10 mm
- Left ventricular chamber diameter >3.0 cm (note: agonal dilatation should be excluded by examining for cell separation and other postmortem artifact histologically)
- If absolute chamber diameter not measured, then comments about gross chamber dilation (without agonal dilatation from autolysis)
- Histologically, myocyte hypertrophy with variable interstitial fibrosis (usually pericellular-type)

Autopsy-negative sudden unexplained death

• Normal heart pathologically with no obvious explanation for death (presumed arrhythmia)

Myocarditis related

• Active lymphocytic myocarditis
- Inflammatory infiltrates of the myocardium with associated myocyte injury/necrosis

• Borderline myocarditis<br>- Inflammatory infiltrates of the myocardium without associated myocyte injury/necrosis.<br>• Healed myocarditis
Coronary artery abnormalities
• Coronary artery anomalies<br>• Myocardial bridging<br>• Tunneled coronary arteries<br>• Coronary dissections
Coronary artery disease
• Atherosclerotic coronary arteries >70% lumen occlusion (thought to be most likely cause of SCD)
Commotio cordis
• SCD after blunt trauma to the chest without other cardiac pathology

CM= cardiomyopathy, DCM= dilated cardiomyopathy, HCM= hypertrophic cardiomyopathy, LV= left ventricular, LVH= left ventricular hypertrophy, NOS= not otherwise specified, RV= right ventricle, SCD= sudden cardiac death
